# Joint modelling of longitudinal data: a scoping review of methodology and applications for non-time to event data

**DOI:** 10.1186/s12874-025-02485-6

**Published:** 2025-02-17

**Authors:** Rehema K. Ouko, Mavuto Mukaka, Eric O. Ohuma

**Affiliations:** 1 Faculty of Epidemiology and Population Health, London School of Hygiene & Tropical Medicine, London, UK; 2 Centre for Tropical Medicine and Global Health, Nuffield Department of Medicine, University of Oxford, Oxford, UK; 3 Mahidol Oxford Tropical Medicine Research Unit, Faculty of Tropical Medicine, Mahidol University, Bangkok, Thailand

**Keywords:** Joint modelling, Longitudinal data, Non-time-to-event outcomes

## Abstract

**Background:**

Joint models are powerful statistical models that allow us to define a joint likelihood for quantifying the association between two or more outcomes. Joint modelling has been shown to reduce bias in parameter estimates, increase the efficiency of statistical inference by incorporating the correlation between measurements, and allow borrowing of information in cases where data is missing for variables of interest. Most joint modelling methods and applications involve time-to-event data. There is less awareness about the amount of literature available for joint models of non-time-to-event data. Therefore, this review’s main objective is to summarise the current state of joint modelling of non-time-to-event longitudinal data.

**Methods:**

We conducted a search in PubMed, Embase, Medline, Scopus, and Web of Science following the PRISMA-ScR guidelines for articles published up to 28 January 2024. Studies were included if they focused on joint modelling of non-time-to-event longitudinal data and published in English. Exclusions were made for time-to-event articles, conference abstracts, book chapters, and studies without full text. We extracted information on statistical methods, association structure, estimation methods, software, etc.

**Results:**

We identified 4,681 studies from the search. After removing 2,769 duplicates, 1,912 were reviewed by title and abstract, and 190 underwent full-text review. Ultimately, 74 studies met inclusion criteria and spanned from 2001 to 2024, with the majority (64 studies; 86%) published between 2014 and 2024. Most joint models were based on a frequentist approach (48 studies; 65%) and applied a linear mixed-effects model. The random effect was the most commonly applied association structure for linking two sub-models (63 studies; 85%). Estimation of model parameters was commonly done using Markov Chain Monte Carlo with Gibbs sampler algorithm (10 studies; 38%) for the Bayesian approach, whereas maximum likelihood was the most common (33 studies; 68.75%) for the frequentist approach. Most studies used R statistical software (33 studies; 40%) for analysis.

**Conclusion:**

A wide range of methods for joint-modelling non-time-to-event longitudinal data exist and have been applied to various areas. An exponential increase in the application of joint modelling of non-time-to-event longitudinal data has been observed in the last decade. There is an opportunity to leverage potential benefits of joint modelling for non-time-to-event longitudinal data for reducing bias in parameter estimates, increasing efficiency of statistical inference by incorporating the correlation between measurements, and allowing borrowing of information in cases with missing data.

**Supplementary Information:**

The online version contains supplementary material available at 10.1186/s12874-025-02485-6.

## Introduction

 Longitudinal studies collect data over time and can, therefore, be used to monitor changes over time and for determination of individual-level changes and influencing factors [1]. To determine change over time in different fields of research, longitudinal study designs are usually employed [1]. However, analysing data from longitudinal studies can be complex [2]. For example, longitudinal data are multidimensional and can have a complex random-error structure that needs to be accounted for during analysis [1]. Challenges related to missing data and losses to follow-up are also common [3, 4]. Most studies are likely to have multiple outcomes that are often correlated and measured repeatedly over time. Multiple outcomes of interest that are correlated and vary over time, should not be modelled separately. Instead, a joint modelling approach that accounts for the correlation and how the underlying relationship changes over time should be considered [5].

Joint models are a powerful class of statistical models that allow us to define a joint likelihood for the quantification of the association between two outcomes [6, 7]. In the last ten years, application of joint modelling for longitudinal data has increased, especially for time-to-event data. Joint modelling has been shown to; a) reduce bias in parameter estimation, b) increase the efficiency of statistical inference by incorporating the correlation between measurements, and c) allow the borrowing of information in cases where there is missing data for one variable of interest [8–11]. Most joint modelling methods and applications are for time-to-event data [12]. However, in practice, two or more time-varying variables are common e.g., fetal head circumference and fetal abdominal circumference associated with an outcome such as small for gestational age or fetal growth restriction. These type of data that are non-time to event are common, and they have not been given much attention in joint modelling. Therefore, there is a need to summarise the current state of joint modelling with applications to non-time-to-event longitudinal data for future research.

The primary aim of this paper is to perform a scoping review of the methodology and applications of joint modelling of non-time-to-event longitudinal data, highlight any gaps and challenges in application of joint modelling for such data, and opportunities to promote their uptake. The specific focus is to review the methods and approaches used for joint modelling of non-time to event data, methods for accounting for the correlation structure, and association structure. Finally, to draw recommendations for future research in this area.

## Methods

The scoping review was conducted according to the Preferred Reporting Items for Systematic Reviews and Meta-Analyses extension for scoping reviews (PRISMA-ScR) checklist [13, 14]. We searched five major databases, Medline, PubMed, Scopus, Embase and Web of Science, for articles published up to 28 January 2024. Studies were included if they reported on joint modelling of longitudinal data for non-time-to-event data. Only studies published in English were included due to the language constraints of the review team. Time-to-event articles were excluded from the review. Furthermore, conference abstracts, book chapters and studies for which no full text is available were also excluded.

**Table:** 

joint model OR joint models OR joint modelling OR joint modeling AND longitudinal OR multivariate OR multivariable OR multilevel OR nonlinear AND NOT (time-to-event* or survival or recurrent event* or competing risk*).	

### Search strategy

A detailed and customised search strategy is available in the supplementary materials as additional file 1.

**Fig. 1:**
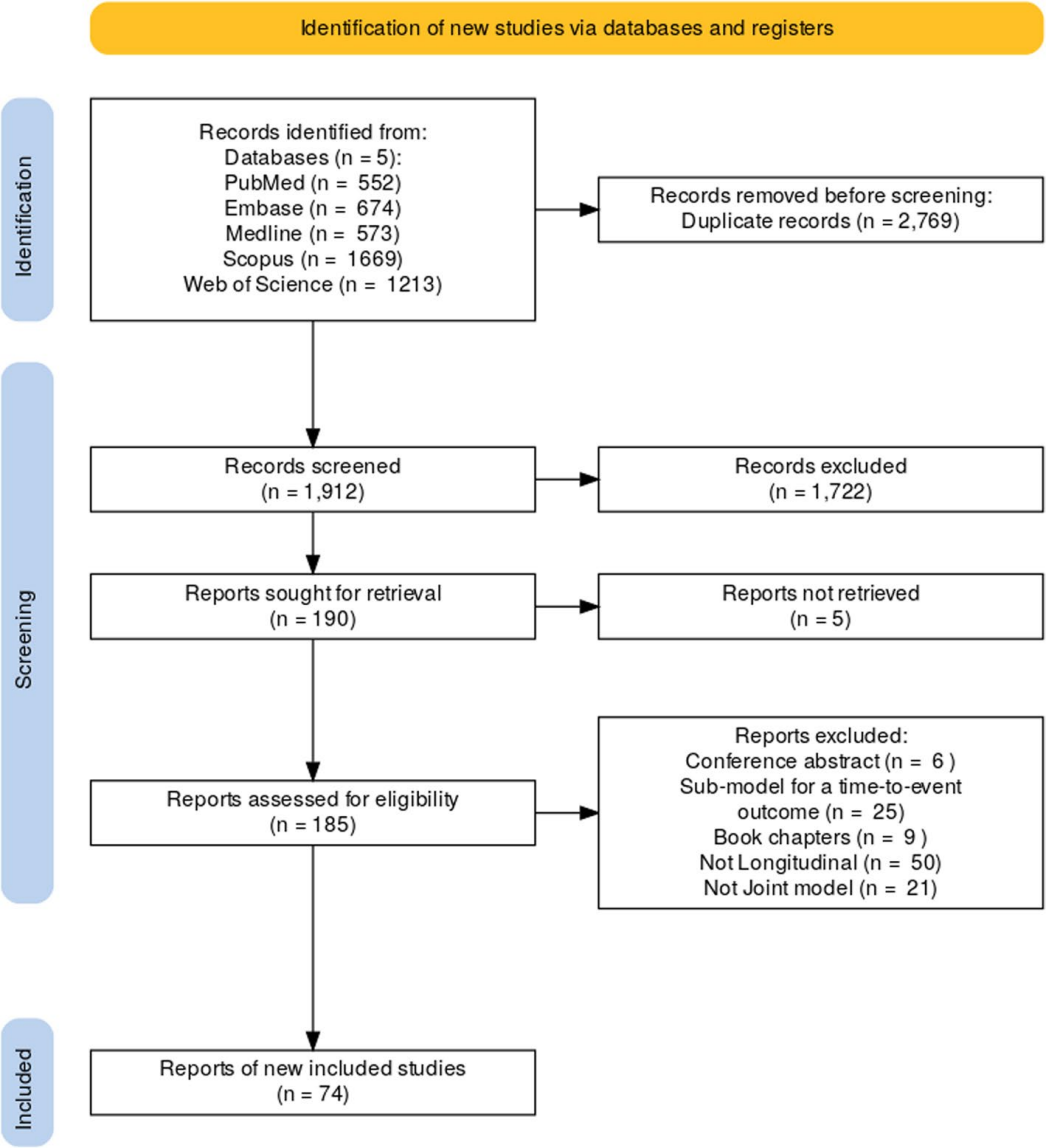
Preferred Reporting Items for Systematic Review Meta-Analysis-Extension for Scoping Reviews flow diagram of search results and study inclusion process

 To sort and manage the articles identified by the search, all articles were imported into Research Information Systems, EndNote Citation Manager, and Rayyan softwares. Duplicates were subsequently removed. The screening process involved screening of article title and abstracts first, and then full-text screening for those passing the title and abstract screening stage. The same reviewer (RKO) independently assessed the full-text version of the eligible articles (Fig. 1). In instances where RKO was uncertain about the inclusion or exclusion of an article, deliberations were conducted with EOO and MM to reach a consensus.

### Data extraction

The articles included were reviewed and data was extracted into a Microsoft Excel template. Information extracted included: publication year, author, journal in which the research was published, the type of longitudinal variables, the sharing structure between the longitudinal sub-models, the type of sub-models used (e.g., linear mixed models, generalised estimating equations), the error distribution, random effects distribution, type of outcome, the statistical methods employed (Bayesian or frequentist), method of estimation, and the specific software used for analysis (e.g., R, SAS, STATA).

## Results

We identified a total of 4,681 from the search. Of these, 2,769 duplicates were identified and removed, 1,912 articles were reviewed at the title and abstract stage form which 190 underwent a full text review. After a full-text review, 74 articles met the inclusion criteria (Fig. 1) and were published between 2001 and January 2024. Articles excluded were conference abstracts, book chapters, and non-English articles.

**Fig. 2:**
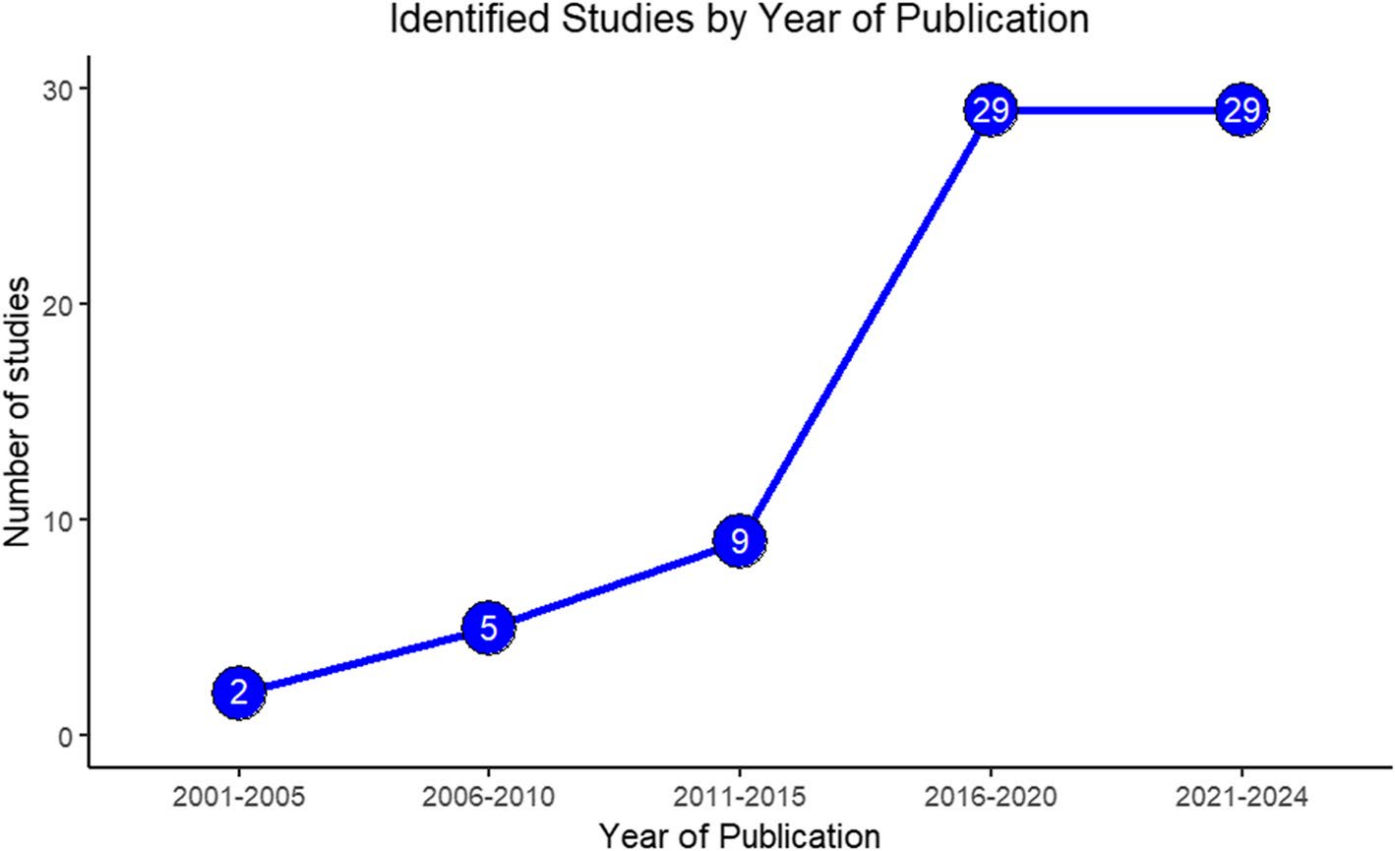
Year of publication of identified studies

 Figure 2 shows the number of studies identified and included in the review by year categorised into five-year time periods. Majority of included studies (*n* = 58, 78%) have been published in the last eight years and have tripled since 2016. The identified studies were published in various academic journals, notably, thirteen specific journals were identified to have published multiple joint modelling studies, with statistics in medicine leading with 13.5% (10 studies).

 Most studies simultaneously analysed two (*n* = 57, 77.0% studies), three (*n* = 13, 17.6% studies) and four (*n* = 3, 4.1% studies) longitudinal variables. One study (*n* = 1, 1.4%) examined the joint modelling of eight mixed longitudinal covariates [15]. Of the total, 26 studies (35%) had longitudinal covariates of the same data type (i.e., either continuous-continuous, binary-binary, count-count, and ordinal-ordinal) with continuous-continuous being the most common (21/26 studies). For those with mixed data type, the majority had a combination of continuous and binary covariates (17/48 studies) (Table 1 in supplementary material additional file 2).

### Longitudinal sub-models

 In general, fifty different sub-models were used where most commonly was linear mixed effect model (LME) for continuous longitudinal covariates (*n* = 54 studies) with three studies applying an adjusted form of LME i.e., finite mixture LME, flexible LME, and flexible spline-based LME model. Generalised linear mixed model (*n* = 43 studies), and hurdle model (*n* = 7 studies). The other 47 models were used infrequently (*n* < 4) (Table 2 in supplementary material additional file 2).

We identified studies (*n* = 10 studies) that utilized nonlinear mixed effects models within the joint modelling framework. The various ways they used to handle nonlinearity include: a semi-parametric quantile regression model with latent variables and threshold parameters [16]; skewed multivariate random effects and a skewed generalized t-link [17]; splines for nonlinear covariate effects [18]; nonparametric priors and splines for flexible covariate modeling; regression splines within a linear mixed effects model [19]; a nonlinear heteroscedastic mixed model based on the Jenss-Bayley growth function [20]; nonlinear mixed models for biomarker profiles [21]; a mechanistic nonlinear model within a logistic regression framework [22]; bivariate thin plate spline surfaces [23]; and a flexible spline-based linear mixed effects model [24].

In the generalised linear mixed models, different link functions were considered: Poisson (6 out of 43 studies), probit (10 out of 43 studies), lognormal (7 out of 43 studies), logit (16 out of 43 studies), and gamma (2 out of 43 studies).

### Error distribution

The random effects were mostly assumed to follow a normal or multivariate normal distribution (*n* = 57, 77%). Similarly, for the distribution of errors, the normal or multivariate normal distributions were commonly assumed (*n* = 50, 68%) (Table 1 in supplementary material additional file 2).

### Association structure

Table 1 summarises the association structures utilised in the different studies. Most of the articles were based on random effects with 36.5% (*n* = 27 studies) using separate random effects, 28.4% (*n* = 21 studies) used random effects but were unspecified whether they were separate or shared, and 21.6% (*n* = 16 studies) were based on a shared random effect.

**Table 1:** Association structure for the joint model

Association Structure for Joint Model	*N* (%)	References
Separate random effects	27 (36.48)	[15, 16, 18, 19, 24–46]
Random effects (unspecified)	21 (28.37)	[20–22, 47–64]
Shared random effects	16 (21.62)	[17, 23, 65–78]
Association parameter	3 (4.05)	[79–81]
Separate and shared random effects	3 (4.05)	[82–84]
Marginal model	1 (1.35)	[85]
Cluster-specific random effect	1 (1.35)	[86]
Separate random effects and Current value association structure	1 (1.35)	[87]
None	1 (1.35)	[88]

### Modelling approach and estimation method

Of the 74 included studies, 48 (64.9%) used a frequentist approach. The most common method of estimation in the frequentist approach was the maximum likelihood (*n* = 33 studies, 68.7%). Other estimation approaches used were: iterative estimation procedure, two-stage estimation procedure, partial marginalisation, and likelihood approaches such as pseudo-likelihood and penalised quasi-likelihood. A Bayesian approach was used by 26 studies (35.1%), with most studies using the Markov Chain Monte Carlo with Gibbs sampler algorithm for estimation (10 of 26 studies, 38%). A variety of techniques were used to assess the convergence of the MCMC chain. Of the 26 studies employing a Bayesian approach, 10 studies (38%) used trace plots or the Gelman-Rubin diagnostics tests (Table 2).

**Table 2:** Method of estimation

Modelling Approach	Method of Estimation	*N* (%)	References
Bayesian	Markov Chain Monte Carlo (Gibbs sampler and Metropolis-Hastings algorithm)	10 (38.46)	[19, 24, 45, 49, 57, 62, 65–67, 70]
	Markov Chain Monte Carlo (Gibbs sampler)	10 (38.46)	[16–18, 22, 43, 46, 58, 68, 69, 80]
	Markov Chain Monte Carlo	4 (15.38)	[20, 30, 36, 44]
	Markov Chain Monte Carlo (Metropolis-Hastings algorithm)	1 (3.84)	[81]
	Hamiltonian Monte Carlo	1 (3.84)	[72]
Frequentist	Maximum likelihood	33 (68.75)	[21, 23, 25, 28, 29, 31–33, 38, 40–42, 48, 50, 52, 53, 55, 56, 60, 61, 63, 64, 71, 73, 76–78, 82–84, 86, 87]
	Likelihood approach	9(18.75)	[15, 27, 34, 35, 37, 47, 51, 54, 59]
	Two-stage estimation procedure	2 (4.16)	[79, 88]
	Iterative estimation procedure	1 (2.08)	[85]
	Partial marginalisation for parameter estimation	1 (2.08)	[26]
	Unclear	3 (6.25)	[39, 74, 75]

### Bayesian priors

Most studies assumed a weak or non-informative prior for the fixed effects, random effects, and association parameters. Priors based on the assumption of a normal distribution were mostly applied to fixed effects (15 of 26 studies, 58%) and random effects (9 of 26 studies, 35%). The Inverse Wishart distribution was the most commonly assumed distribution for the association structures (21 of 26 studies, 81%) (Table 1). Sensitivity analysis to evaluate the impact of the assumed priors was performed in only half of the studies (13 of 26 studies, 50%) (Table 3).

**Table 3:** Bayesian modelling approach priors

Bayesian Modelling Approach Priors		*N* (%)	References
Fixed Prior	Normal	15 (57.69)	[16–18, 20, 24, 36, 44, 45, 58, 62, 68–70, 80, 81]
	Multivariate normal	3 (11.53)	[46, 49, 66]
	Half Cauchy distribution	1 (3.85)	[72]
	P variate normal	1 (3.85)	[65]
	Shrinkage prior normal	1 (3.85)	[43]
	Unclear	5 (19.23)	[19, 22, 30, 57, 67]
Random effects Prior	Normal	9 (34.62)	[16–18, 45, 46, 62, 68–70]
	Multivariate normal	3 (11.53)	[36, 49, 66]
	Dirichlet process prior	3 (11.53)	[19, 24, 43]
	P variate normal	1 (3.84)	[65]
	Log-normal distribution	1 (3.84)	[44]
	Bivariate normal distribution	1 (3.84)	[58]
	Half Cauchy distribution	1 (3.84)	[72]
	Inverted Gamma prior	1 (3.84)	[57]
	Unclear	6 (23.08)	[20, 22, 30, 67, 80, 81]
Association Parameter Prior	Inverse Wishart	21 (80.76)	[16–20, 24, 36, 43–46, 49, 58, 62, 65–70, 80]
	Normal distribution	1 (3.44)	[81]
	Unclear	4 (15.38)	[22, 30, 57, 72]

### Software

R statistical software was the most used (*n* = 33, 45%) followed by SAS (*n* = 25 studies, 34%). Other softwares used included: WinBUGs, Fortran, MATLAB, Stata, and SPSS. Nine of the studies (12%) did not report the software used (Table 4).

**Table 4:** Software used in joint modelling

Software	No. (%)	References
R	33 (44.59%)	[16, 19, 20, 22–24, 27, 29, 30, 36, 38, 40, 42–44, 46, 47, 49, 52, 56, 58, 63–66, 68, 70–73, 76–78]
SAS	25 (33.78%)	[15, 21, 26, 28, 32–34, 37, 39, 40, 42, 48, 50, 51, 53, 55, 56, 59, 60, 71, 74, 83–86]
WinBUGS	8 (10.81%)	[18, 43–45, 62, 67, 69, 81]
Fortran	1 (1.35%)	[17]
MATLAB	2 (2.70%)	[57, 61]
Stata	3 (4.05%)	[35, 56, 87]
SPSS	1 (1.35%)	[77]
Unclear	9 (12.16%)	[25, 31, 41, 54, 75, 79, 80, 82, 88]

## Discussion

Several approaches have been proposed and employed to jointly model longitudinal and non-time-to-event data. The frequentist method was the most used modelling approach for longitudinal data using a linear mixed-effects model. The association structure for linking the two models was mostly a random effect. Markov Chain Monte Carlo with Gibbs sampler algorithm was commonly used to estimate the model parameters under the Bayesian approach and maximum likelihood for the frequentist approach. The most common area of application was in medical research. R software was the most commonly used.

Most studies reviewed utilised Maximum Likelihood Estimation (MLE) as the primary frequentist method for parameter estimation. The MLE approach is favoured for its desirable statistical characteristics, such as consistency and asymptotic normality, which makes it a robust choice for complex joint modelling scenarios [89–91]. MLE provides consistent parameter estimates, meaning that as the sample size increases, the estimates converge to the true parameter values, ensuring the reliability of the model’s predictions [89]. Additionally, MLE estimates are asymptotically normally distributed, allowing for the construction of confidence intervals and hypothesis tests, simplifying the inference process [90]. Furthermore, MLE is efficient, achieving the lowest possible variance among all unbiased estimators under certain conditions and ensuring precise estimates [91].

Despite its advantages, MLE has limitations, such as computational complexity, particularly in high-dimensional contexts or scenarios with complex correlation structures, especially in joint modelling frameworks [15]. However, using parallel computing techniques can help spread the computational workload across multiple processors, significantly reducing the time required to estimate the parameters. Additionally, implementing more efficient algorithms, like the Expectation-Maximization (EM) algorithm, can tackle complex models more effectively [92, 93]. Also, we have methods like the Laplace approximation or variational inference that can help simplify the likelihood function, making the calculations more manageable [10].

Furthermore, MLE depends on the correct specification of the model, and violating model assumptions can lead to biased parameter estimates. It can also be sensitive to outliers, which can significantly affect the parameter estimates, necessitating careful data preprocessing and outlier detection [26]. However, employing flexible models that accommodate a wide range of data distributions and correlation structures, such as semiparametric models, can relax the assumption of normal distribution, for example, in random effects [94]. Furthermore, using robust estimation techniques that are less affected by outliers, such as M-estimators or trimmed likelihood methods, can help ensure robust parameter estimates [95, 96].

Different Bayesian sampling algorithms were applied to those studies that employed the Bayesian approach. The MCMC is generally used to estimate the parameters in the Bayesian approach. The combination of Gibbs sampler and Metropolis-Hastings (MH) algorithms enables faster convergence. Gibbs sampling can quickly explore the parameter space for some variables, while MH can handle the more challenging parts, leading to a more efficient overall sampling process. In high-dimensional spaces, pure MH can struggle with low acceptance rates. Gibbs sampling can break down the problem into lower-dimensional conditional distributions, making it easier for MH to operate effectively on the remaining dimensions [97]. In addition, the combination of Gibbs sampling and MH leads to flexibility in sampling, as the Gibbs Sampler is efficient for sampling from conditional distributions when it is easy to sample directly, and Metropolis-Hastings is useful for sampling from complex distributions where direct sampling is difficult. Hence, Gibbs sampling can be used for variables with straightforward conditional distributions and MH for more complex ones [97].

Assessing the convergence of MCMC when Bayesian estimation is employed is essential. Diagnostic tools have been created to assess the time it takes for the chain to generate observations from the stationary distribution of the Markov chain [98]. The studies reviewed used the Gelman-Rubin diagnostics test, trace plots, autocorrelation plots, cross-correlation plots, density plots, and MCMC chain history.

Bayesian estimation offers a clear advantage by allowing the incorporation of previous studies’ information through prior parameter distributions. A prior is typically defined for the unknown fixed effect parameter and the association parameter. This means that prior information from previous studies is used to influence the posterior distribution [8] Some studies also assume a prior for both fixed and random effect parameters in longitudinal trajectory, providing more flexibility in modelling the trajectory and reducing uncertainty regarding distributional assumptions. The most popular prior used was normal prior, and the Dirichlet process prior was used to enable the creation of a family of distributions for more flexible priors than the standard normal distribution.

One of the advancements of joint modelling is prediction [8, 99]. Of the studies reviewed, few articles provided dynamic predictions for non-time-to-event outcomes. Prediction is beneficial in medical research as it aids in tailoring diseases and conditions for individuals and hence takes a relatively accurate decision to improve the decision-making procedure in health [8]. Hence, there is a potential benefit of using joint modelling for the non-time-to-event longitudinal data, leveraging this advantage among others.

Joint modelling can be more challenging when the longitudinal model is nonlinear [93]. In this review, studies that utilized nonlinear mixed effects models within the joint modelling framework were identified. For instance, the use of semi-parametric quantile regression models, skewed multivariate random effects, and splines for nonlinear covariate effects highlight the diverse applications of these methods [17, 24, 45]. Additionally, the incorporation of nonparametric priors, mechanistic nonlinear models, and penalized splines further illustrates the flexibility of nonlinear approaches in joint modeling [18, 22]. These methodologies not only enhance the accuracy of predictions but also provide a comprehensive understanding of the underlying processes in various research contexts. The findings from these studies underscore the importance of considering nonlinear mixed effects models in joint modeling to address the complexities inherent in longitudinal data analysis [100].

In joint modelling, error distributions play a crucial role in accurately capturing the relationship between different repeated measurements. In most studies, the normal distribution and multivariate normal distributions were commonly used, assuming that the residuals (errors) follow a normal distribution. Two studies [16, 46] used an Asymmetric Laplace distribution to handle different quantiles of the data as they used a quantile regression model. The error distributions help link the longitudinal sub-models, ensuring that the joint model accurately reflects the underlying data structure [8, 12].

Similarly, as the error distributions, the majority of the studies employed normal distribution and multivariate normal distributions for the random effects. Some studies used a skewed normal distribution to address the uncertainties in random effects. Distributional assumptions are vital for parameter estimation and inference [101]. However, mis-specifying the distribution can lead to biased estimates and incorrect inferences [102]. In certain cases, a Dirichlet process prior is assigned to the random effects to provide flexibility and prevent mis-specification of the random effects distribution [103]. We found out that most of the articles were based on random effects as an association structure in the joint model, which links the longitudinal sub-models and allows for individual-specific predictions. The individual-specific information is crucial for describing the outcomes, for instance, disease course, and even designing interventions for the subjects [104]. Some studies specified whether they used separate or shared random effects with separate random effects being the most common. Separate random effects provide more flexibility in modelling as they allow for different sources of variability in both sub-models [12]. This allows a straightforward interpretation of random effects pertaining to a specific sub-model. In some cases, it leads to a better data fit, especially if the correlation between the longitudinal measurements is weak. In addition, separate random effects accommodate more complex association structures between the longitudinal measurements [10]. Shared random effects have the advantage of improving efficiency through borrowing of information that leads to more efficient parameter estimates [8]. It also allows a simplified model structure by reducing the number of parameters that need to be estimated [12]. Choosing between these approaches depends on the specific context of the study and the nature of the data. Shared random effects are often preferred for their efficiency and ability to simplify the model structure, while separate random effects offer greater flexibility and interpretability.

To conclude, we have reviewed studies with Bayesian and frequentist approaches, summarised the modelling approach, type of sub-model, association structure, sampling algorithms, priors employed, the software used, whether a simulation study was conducted, and dynamic predictions.

## Supplementary Information

Supplementary Material 1.

Supplementary Material 2.

## Data Availability

No datasets were generated or analysed during the current study.
