# Supplementary material for: Joint modelling of longitudinal data: a scoping review of methodology and applications for non-time to event data
Source: BMC Med Res Methodol. 2025 Feb 17;25:40. doi: 10.1186/s12874-025-02485-6 (PMC11831847; doi:10.1186/s12874-025-02485-6)
Supplement: Supplementary file 1 — Supplementary Material 1. [file 12874_2025_2485_MOESM1_ESM.docx]

**Joint modelling of longitudinal data: a scoping review of methodology and applications for non-time to event data**

**Additional file 1: Search strategy**

| **Database** | **Search Strategy** |
| --- | --- |
| Embase | 1. "joint model*".ab,hw,kf,ot,ti. |
|  | 2.(longitudinal or Multivar* or non-linear or nonlinear or multilevel).ab,hw,kf,ot,ti. |
|  | 3.1 and 2 |
|  | 4.(time-to-event* or survival or recurrent event* or competing risk*).ab,hw,kf,ot,ti. |
|  | 5. 3 NOT 4 |
| Medline | 1."joint model$".ab,hw,kf,ot,ti. |
|  | 2. (longitudinal or Multivar$ or non-linear or nonlinear or multilevel).ab,hw,kf,ot,ti. |
|  | 3.1 and 2 |
|  | 4.(time-to-event$ or survival or recurrent event$ or competing risk$).ab,hw,kf,ot,ti. |
|  | 5.3 NOT 4 |
| Web of science | 1.TS=("joint model*") |
|  | 2.TS=(longitudinal or Multivar* or non-linear or nonlinear or multilevel) |
|  | 3.#1 and #2 |
|  | 4.TS=(time-to-event* or survival or recurrent event* or competing risk*) |
|  | 5.#3 NOT #4 |
| Scopus | 1.TITLE-ABS-KEY ( "joint model*" ) |
|  | 2.TITLE-ABS-KEY ( ( longitudinal OR multivar* OR non-linear OR nonlinear OR multilevel ) ) |
|  | 3. 1 AND 2 |
|  | 4.TITLE-ABS-KEY ( time-to-event* OR survival OR "recurrent event*" OR "competing risk*" ) |
|  | 5. 3 NOT 4 |
| Pubmed | 1.joint model*[Title/Abstract] |
|  | 2.longitudinal[Title/Abstract] OR Multivar*[Title/Abstract] OR non-linear[Title/Abstract] OR nonlinear[Title/Abstract] OR multilevel[Title/Abstract] |
|  | 3. #1 AND #2 |
|  | 4.time-to-event*[Title/Abstract] OR survival[Title/Abstract] OR "recurrent event*"[Title/Abstract] OR "competing risk*"[Title/Abstract] |
|  | 5. #3 NOT #4 |
