# Supplementary material for: Joint modelling of longitudinal data: a scoping review of methodology and applications for non-time to event data
Source: BMC Med Res Methodol. 2025 Feb 17;25:40. doi: 10.1186/s12874-025-02485-6 (PMC11831847; doi:10.1186/s12874-025-02485-6)
Supplement: Supplementary file 2 — Supplementary Material 2. [file 12874_2025_2485_MOESM2_ESM.docx]

**Joint modelling of longitudinal data: a scoping review of methodology and applications for non-time to event data**

**Additional file 2: Tables**

**Table 1: Characteristics of the longitudinal sub-models**

| **Type of Variables** | **N(%)** |
| --- | --- |
| Continuous | 21 (28.38) |
| Continuous and Binary | 17 (22.97) |
| Continuous and Count | 9 (12.16) |
| Continuous and Ordinal | 8 (10.81) |
| Continuous, Binary and Ordinal | 4 (5.41) |
| Binary and Ordinal | 3 (4.05) |
| Binary | 3 (4.05) |
| Count and Binary | 2 (2.70) |
| Continuous, proportion, binary and Count | 1 (1.35) |
| Count and Categorical | 1 (1.35) |
| Discrete and Continuous | 1 (1.35) |
| Count | 1 (1.35) |
| Ordinal | 1 (1.35) |
| Percentage and Count | 1 (1.35) |
| Semi-continuous and Continuous | 1 (1.35) |
| **Error distribution** |  |
| Normal distribution | 44 (59.46) |
| Multivariate normal | 6 (8.11) |
| Asymmetric Laplace | 2 (2.70) |
| Skew-normal distribution | 2 (2.70) |
| Normal, skewed-normal, skewed-t distributions | 1 (1.35) |
| Skewed-t distribution, Asymmetric Laplace distribution | 1 (1.35) |
| Unspecified | 18 (24.32) |
| **Random effect distribution** |  |
| Normal distribution | 30 (40.54) |
| Multivariate normal | 27 (36.48) |
| Bivariate normal distribution | 3 (4.05) |
| No random effects | 3 (4.05) |
| Normal, Dirichlet process | 1 (1.35) |
| Dirichlet process | 1 (1.35) |
| Gaussian distribution | 1 (1.35) |
| Normal distribution, Bridge distribution | 1 (1.35) |
| Skew-normal distribution | 1 (1.35) |
| Unspecified | 6 (8.11) |

**Table 2: Summary of the longitudinal sub-models**

| **Longitudinal Sub-models** | **N (%)** |
| --- | --- |
| Linear mixed effects model | 54 (31.95) |
| Generalized linear mixed model | 43 (25.44) |
| Hurdle model | 7 (4.14) |
| Generalized estimating equations | 4 (2.36) |
| Polynomial regression spline | 3 (1.77) |
| Beta mixed model | 2 (1.18) |
| Inverse Probability Treatment Weighting-weighted logistic regression | 2 (1.18) |
| Marginalized random-effects model | 2 (1.18) |
| Mixed-effect models | 2 (1.18) |
| Mixed-effects location scale model | 2 (1.18) |
| Multinomial logit model | 2 (1.18) |
| Multiple inflated negative binomial regression model | 2 (1.18) |
| Nonlinear mixed‐effects model (Jenss‐Bayley growth function) | 2 (1.18) |
| Quantile regression model | 2 (1.18) |
| Semi parametric mixed effects model (smooth functions) | 2 (1.18) |
| Semi-parametric quantile regression model with cubic spline | 2 (1.18) |
| Ecological regression model with random effects | 2 (1.18) |
| Semi varying coefficient model | 2 (1.18) |
| Correlated probit model with random effects | 2 (1.18) |
| Logistic Regression with random effects | 2 (1.18) |
| A transitive cumulative logistic regression model | 1 (0.59) |
| A-inflated normal regression model | 1 (0.59) |
| Autoregressive regression model | 1 (0.59) |
| B -inflated power series regression model | 1 (0.59) |
| Binary logit model | 1 (0.59) |
| Covariate measurement error model | 1 (0.59) |
| Hierarchical Gaussian process model | 1 (0.59) |
| Local linear regression | 1 (0.59) |
| Log-normal model | 1 (0.59) |
| LogLindley-Binomial model | 1 (0.59) |
| LogLindley-Ordinal model | 1 (0.59) |
| Logistic model with the cumulative probability | 1 (0.59) |
| Mechanistic Nonlinear Model with Random Effects | 1 (0.59) |
| Mixed effects spline regression | 1 (0.59) |
| Mixed-effects location scale Tobit model | 1 (0.59) |
| Mixed-effects logistic regression model | 1 (0.59) |
| Negative-binomial mixed model | 1 (0.59) |
| Penalized spline model | 1 (0.59) |
| Poisson mixed effects regression | 1 (0.59) |
| Quantile regression-based partially linear mixed-effects model | 1 (0.59) |
| Random intercept model | 1 (0.59) |
| Random intercept–slope model | 1 (0.59) |
| Semi-parametric semi-continuous model | 1 (0.59) |
| Skew-normal mixed effects model | 1 (0.59) |
| Standard correlated regression model | 1 (0.59) |
| Two-part models | 1 (0.59) |
| Zero-Inflated Beta Binomial-Normal, Zero-Inflated LogLindley-Normal | 1 (0.59) |
| Zero-Inflated Beta Binomial, Zero-Inflated LogLindley-Binomial | 1 (0.59) |
| Log Lindley-binomial Normal Mixed Model, Beta-binomial mixed model | 1 (0.59) |
| Zero-inflated binomial model | 1 (0.59) |
